# Brain Levels of Prostaglandins, Endocannabinoids, and Related Lipids Are Affected by Mating Strategies

**DOI:** 10.1155/2013/436252

**Published:** 2013-11-27

**Authors:** Jordyn M. Stuart, Jason J. Paris, Cheryl Frye, Heather B. Bradshaw

**Affiliations:** ^1^Department of Psychological and Brain Sciences, Indiana University, 1101 East 10th Street, Bloomington, IN 47405, USA; ^2^Department of Psychology, The University at Albany, SUNY, 1400 Washington Ave, Albany, NY 12222, USA; ^3^Department of Chemistry, University of Alaska-Fairbanks, 900 Yukon Drive, Fairbanks, AK 99775-6160, USA

## Abstract

*Background*. Endogenous cannabinoids (eCBs) are involved in the development and regulation of reproductive behaviors. Likewise, prostaglandins (PGs) drive sexual differentiation and initiation of ovulation. Here, we use lipidomics strategies to test the hypotheses that mating immediately activates the biosynthesis and/or metabolism of eCBs and PGs and that specific mating strategies differentially regulate these lipids in the brain. *Methods*. Lipid extractions and tandem mass spectrometric analysis were performed on brains from proestrous rats that had experienced one of two mating strategies (paced or standard mating) and two nonmated groups (chamber exposed and home cage controls). Levels of PGs (PGE2 and PGF2alpha), eCBs (AEA and 2-AG, *N*-arachidonoyl glycine), and 4 related lipids (4 *N*-acylethanolamides) were measured in olfactory bulb, hypothalamus, hippocampus, thalamus, striatum, midbrain, cerebellum, and brainstem. *Results*. Overall, levels of these lipids were significantly lower among paced compared to standard mated rats with the most dramatic decreases observed in brainstem, hippocampus, midbrain, and striatum. However, chamber exposed rats had significantly higher levels of these lipids compared to home cage controls and paced mated wherein the hippocampus showed the largest increases. *Conclusions*. These data demonstrate that mating strategies and exposure to mating arenas influence lipid signaling in the brain.

## 1. Introduction

Decades of studies on mating behavior in laboratory rats (typically *Rattus norvegicus*) provide a wealth of knowledge about developmental and motivational roles of various neurotransmitter systems in sexual differentiation and/or manifestation of reproductive behaviors [1]. Standard mating procedures for rats in laboratory environments typically involve placing a sexually experienced male rat in a testing chamber (these vary in size but are usually around 60 × 50 × 40 cm aquaria) with a female in behavioral estrus (the time of ovulation and sexual receptivity in female rats). In this situation, typically rats mate with the timing of sexual contacts being driven by the male until he ejaculates. When mating chambers are large enough, males often participate in a common preintercourse sequence of sniffing, hopping, and then mounting [2]. In this context, these behaviors are both initiated and regulated by the male in that the male is able to “pace” his interactions with the female, which may engender intrinsic reward, associating the females as a conditioned incentive [2]. Although this standard procedure for mating is rewarding for males, female rats that cannot pace their sexual interactions typically do not develop a conditioned place preference as opposed to their female counterparts that “pace” their sexual contacts in a mating strategy called paced mating [2, 3]. In the laboratory, “paced mating boxes” are larger mating arenas, which have a divider with a small hole. This apparatus allows females to engage and withdraw from males and have some control over the receipt of copulatory stimuli. This paradigm has been used to ascertain neurophysiological mechanisms associated with paced mating and/or standard mating (partition is removed).

Endocannabinoids are endogenous lipid neurotransmitters that activate cannabinoid receptors and play a role in regulating motivated behaviors, such as feeding, anxiety, drug seeking, pain, and reproduction [4, 5]. The most studied of the endogenous ligands are *N*-arachidonoyl ethanolamine (anandamide; AEA), 2-arachidonoyl glycerol (2-AG) [6], and more recently the endogenous metabolite of AEA *N*-arachidonoyl glycine (NAGly) was shown to activate the GPR18, which is a putative cannabinoid receptor [7–9]. Cannabinoid agonist, WIN 55,212-2 (WIN), administered to male rats reduced intromission frequency and increased intervals between ejaculations [4]. Injecting proestrus, but not hormone primed, rats with a CB_1_ (cannabinoid receptor 1) antagonist/inverse agonist and GPR18 antagonist, AM251 facilitated sexual motivation [4]. Levels of endocannabinoid ligands (AEA, NAGly, and 2-AG) change significantly in rodent brain with the estrous cycle and show sex differences, suggesting a preparatory role for mating [10]. Indeed, progesterone can also upregulate CB_1_ receptor activity in the hypothalamus [11]. Together these findings suggest a mutual regulation between the endocrine system and endocannabinoid system which may play a role in the neuronal control of mating and its rewarding properties.

A structurally similar lipid signaling system to the endocannabinoids and the prostaglandins, specifically PGE2, may act in the hypothalamus by inhibiting release of a prolactin-secretion-inhibiting factor during mating, which could contribute to induction of prolactin surges [12]. PGE2 can facilitate lordosis in response to mounting among estrogen primed, ovariectomized, and adrenalectomized rats [13]. In paced mating paradigms, the interval between sexual contacts is directly related to how much stimulation females receive and increases with each encounter [14]. Among female guinea pigs (also spontaneous ovulators), prostaglandin release in response to mating may disrupt hypothalamus stimulatory norepinephrine signaling, which leads to the postmating inhibition of sexual behavior [12]. Prostaglandins are key components in the mechanism leading up to the follicular rupture involved in ovulation at the site of the ovary [15]. Their influence is demonstrated at the level of the hypothalamus and pituitary by releasing luteinizing hormone (LH), a gonadotropin essential for the onset of ovulation [15, 16].

Lipidomics techniques, in which lipid extracts from tissues are analyzed using tandem mass spectrometry, allow us to measure multiple different lipids from the same tissue and determine relative amounts of lipid between brain areas and treatment groups. Here, we test the hypothesis that production of the prostaglandins PGE2 and PGF_2*α*_, as well as the endocannabinoid ligands AEA, 2-AG, and NAGly, and structurally related lipids and signaling molecules *N*-palmitoyl ethanolamine (PEA), *N*-oleoyl ethanolamine (OEA), N-docosahexaenoyl ethanolamine (DHEA), and *N*-stearoyl ethanolamine (SEA) are differentially regulated acutely by mating strategies in the female rodent brain. Brains from female rats that were either paced or standard mated and two control groups (chamber exposed and home cage control) were analyzed using high-performance liquid chromatography tandem mass spectrometry (HPLC/MS/MS) for production levels of the lipids listed above in eight different brain regions (olfactory bulb, hypothalamus, hippocampus, thalamus, striatum, midbrain, cerebellum, and brainstem). Overall, levels of these lipids were significantly lower among paced compared to standard mated rats in the majority of brain areas with the most dramatic decreases observed in brainstem, hippocampus, midbrain, and striatum. However, chamber exposed rats had significantly higher levels of these lipids than did home cage controls wherein the hippocampus showed the largest increases. These data demonstrate that mating strategies and exposure to mating arenas influence lipid signaling in the brain and imply that eCBs, *N*-acylethanolamines, and PGs are involved in driving the neurophysiological outcomes of mating behaviors.

## 2. Methods

### 2.1. Materials

Arachidonoyl ethanolamide-d4 (d4-AEA) was purchased from Tocris Bioscience (St. Louis, MO). AEA, PEA, SEA, OEA, DHEA, and 2-AG were purchased from Cayman Chemical (Ann Arbor, MI). NAGly was purchased from Biomol (Plymouth Meeting, PA). HPLC-grade water and methanol were purchased from VWR International (Plainview, NY). HPLC-grade acetic acid and ammonium acetate were purchased from Sigma-Aldrich (St. Louis, MO).

#### 2.1.1. Animals—Animal Subjects Used in This Experiment Were Housed at the State University of New York (SUNY) Albany

Age-matched and littermate female Long-Evans rats (4–6 months old) in behavioral estrus (*n* = 6 per group) and sexually experienced male rats were maintained on a 12 : 12 h reversed dark-light cycle (08:00 dark and 18:00 light). Food and water were available *ad libitum*. Vaginal cytology of rats was obtained daily to assess phase of the estrous cycle. On the day of testing, all females (even control groups) with a proestrous smear were vaginally masked and were behaviorally assessed with a male to make sure they were sexually receptive, which was determined by responding to one male mount with lordosis. Only sexually receptive females were used as test subjects. They were then returned to their home cages for a minimum of 2.5 hours before testing.

*Experimental Conditions.* This protocol was according to Erskine, 1985 [14]. It was performed at the SUNY Albany in the Laboratory of Cheryl Frye.

Animals were tested during the dark phase of the cycle between the hours of 08:00 and 16:00. The animals were transported from the animal housing room to the testing area in their home cages, where they were placed outside the testing room on a rack until testing began. Experimental subjects had vaginal masks affixed to the perineum to minimize mating-induced changes. Behavioral analyses and manipulations were taken by an observer who was unaware of the hypotheses and experimental conditions.

### 2.2. Paced Mating

Testing was conducted in a white melamine chamber (37.5 × 75 × 30 cm) that was divided into two compartments via a Plexiglas divider that had been cleaned with quatricide and allowed to dry. This apparatus was also constructed and tested to confirm it was functioning properly before testing began. The Plexiglas divider had a small (5 cm) hole in the bottom center that was large enough for a female to pass through but not large enough for a male. The males had also been previously conditioned to stay away from the hole. This allowed the females to self-administer or “pace” their mating by controlling the frequency of mating contacts and the amount of time between mounts, intromissions, and ejaculation. The males were habituated to the testing chamber first, followed by the female in the opposite side of the chamber. Their sexual interaction was observed for 15 minutes or until the first ejaculatory emission was reached. Lordosis quotients, aggression quotients, proceptivity, and percent exits were measured. Once the fifteen minutes or the first ejaculatory emission was reached, the female was immediately removed from the chamber and decapitated. The brain was then removed and immediately frozen on dry ice. The chamber was then cleaned with quatricide and allowed to dry before the next trial was done.

### 2.3. Standard Mating

The same 37.5 × 75 × 30 cm melamine chamber that was used for paced mating was also used for this test group. The Plexiglas divider was removed for this experiment. This allowed the male, instead of the female, to control how often mating was administered. The females were vaginally masked to prevent pregnancy and other mating-induced changes.

### 2.4. Chamber Exposed

A female was placed in the 37.5 × 75 × 30 cm melamine chamber with the Plexiglas divider (same as paced mating chamber design) inserted, for 15 minutes. She was then immediately removed from the chamber and decapitated. The brain was removed and immediately flash-frozen on dry ice. The chamber was then cleaned with quatricide and allowed to dry before the next trial was done.

### 2.5. Home Cage Control

Females were taken from their housing chambers and decapitated. The brains were removed and immediately flash-frozen on dry ice. They had no social exposure (to males) and no chamber exposure.

### 2.6. Tissue Dissection

After all the tissues were collected, the tissue was sent overnight on dry ice from Albany, NY, to Bloomington, IN, where it was stored in a −80°C freezer until brains were dissected and processed. Tissue dissection and storage were performed as previously described by Bradshaw et al., 2006 [10]. In brief, the frozen brains were thawed for approximately 5 minutes on an ice cold tin foil covered dissection plate. Once thawed, brains were dissected into the following regions: olfactory bulb, hypothalamus, striatum, thalamus, hippocampus, midbrain, brainstem, and cerebellum. Each region was then placed in a 1.5 mL microfuge tube and flash-frozen with liquid nitrogen. They were stored in the −80°C freezer until used for lipid extractions.

### 2.7. Lipid Extraction

Each brain area was processed separately and all tissues from a specific brain area were processed together, although the order of processing was randomized, as previously described [10]. The samples were removed from the −80°C freezer. After being shocked with liquid nitrogen, they were weighed and placed in centrifuge tubes on ice. Furthermore, 40 : 1 volumes of methanol were added to each tube followed by 10 *μ*L of 1 uM d4-AEA. d4-AEA was added to act as an internal standard to determine the recovery of the compounds of interest. The tubes were then covered with parafilm and left on ice and in darkness for approximately 2 hours. Remaining on ice, the samples were then homogenized using a polytron for approximately 1 minute on each sample. The samples were then centrifuged at 19,000 ×g at 24°C for 20 minutes. The supernatants were then collected and placed in polypropylene tubes (15 or 50 mL), and HPLC-grade water was added making the final supernatant/water solution 25% organic. To isolate the compounds of interest, partial purification of the 25% solution was performed on a Preppy apparatus (Sigma-Aldrich) assembled with 500 mg C18 solid-phase extraction columns (Agilent Technologies, Santa Clara, CA). The columns were conditioned with 5 mL of HPLC-grade methanol immediately followed by 2.5 mL of HPLC-grade water. The supernatant/water solution was then loaded onto the C18 column and then washed with 2.5 mL of HPLC-grade water followed by 1.5 mL of 40% methanol. The prostaglandins were then collected with a 1.5 mL elution of 70% methanol, NAGly with a 1.5 mL elution of 85% methanol, and the ethanolamides with a 1.5 mL elution of 100% methanol. All were collected in individual autosampler vials and then stored in a −20°C freezer until mass spectrometer analysis.

### 2.8. LC/MS/MS Analysis and Quantification

**Figure 1:**
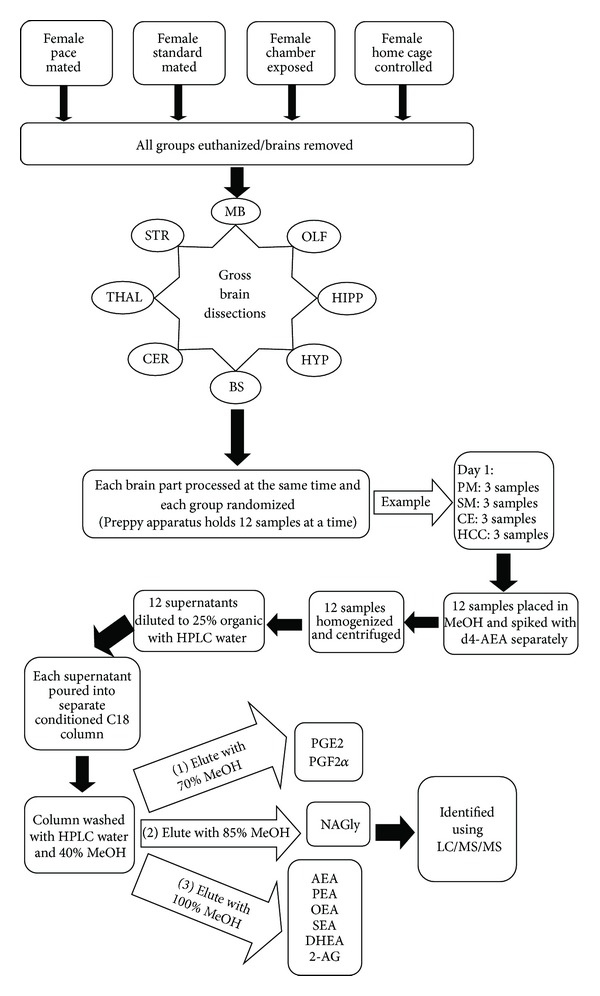
Flowchart of experimental design and lipidomic methodology—starting with the 4 different mating strategies treatment groups, euthanization and collection of brains, gross brain dissection, and then the randomization of the lipid extraction process to the standardized elution and HPLC/MS/MS analysis. See Section 2 for abbreviation definitions.

Samples were removed from the −20°C freezer and allowed to warm to room temperature and then vortexed for approximately 1 minute before being placed into the autosampler and held at 24°C (Agilent 1100 series autosampler, Palo Alto, CA) for LC/MS/MS analysis. Also 10–20 *μ*L of eluants was injected separately for each sample to be rapidly separated using a C18 Zorbax reversed-phase analytical column (Agilent Technologies, Santa Clara, CA) to scan for individual compounds (mobile phase A: 20% HPLC methanol, 80% HPLC water, and 1 mM ammonium acetate; mobile phase B: 100% HPLC methanol and 1 mM ammonium acetate). Gradient elution (200 *μ*L/min) then occurred under the pressure created by two Shimadzu 10AdVP pumps (Columbia, MD). Next, electrospray ionization was accomplished using an Applied Biosystems/MDS Sciex (Foster City, CA) API3000 triple quadrupole mass spectrometer. A multiple reaction monitoring (MRM) setting on the LC/MS/MS was then used to analyze levels of each compound present in the sample injection. Synthetic standards were used to generate optimized MRM methods and standard curves for analysis.  Figure 1 shows a flowchart of the extraction process starting after the animals had been mated.

### 2.9. Data Analyses

The amount of analyte in each sample was calculated by using a combination of calibration curves of the synthetic standards and deuterium-labeled internal standards obtained from the Analyst software. The standards provided a reference for the retention times by which the analytes could be compared. They also helped to identify the specific precursor ion and fragment ion for each analyte which enabled their isolation. These processes provide confidence in the claim that the compounds measured were, in fact, the compounds of interest. The amount of each compound in each tissue was then converted to moles per gram tissue, which is how it was statistically analyzed.

The current study had 4 treatment groups (*n* = 6/grp) and profiled 9 lipids in 8 different brain regions generating over 1700 data points. In an effort to consider the relatedness between analytes in each brain region, general linear models (GLM) were used to consider the experimental conditions between subjects variables and analytes as nested variables across brain regions.  Table 1 summarizes the *P* values from the GLM analysis for each brain region. Using this analysis, it was shown that there were significant interactions between analyte and treatment group in the brainstem (BS), hippocampus (HIPP), midbrain (MB), and striatum (STR). Therefore, post hoc analyses of each individual group to each other were performed using ANOVA, described below. Using this criterion, additional post hoc analyses were not performed on the remaining four brain regions analyzed. Those values are presented in Table 2.

Data from the BS, HIPP, MB, and STR were subsequently analyzed for each brain using the nontested group as the control value compared to the chamber exposed, standard mated, and paced mated group. Follow-up analyses considered the chamber exposed group as the experimental control compared to standard and paced mated groups. Finally, the standard mated group data was compared to the paced mated group. Each comparison was a one-way ANOVA with post hoc Fisher's LSD with a 95% confidence interval for the mean using SPSS software. Data in Tables 3–6 are presented as means ± SE of the means, where *P* ≤ 0.05 was considered statistically significant.

## 3. Results

All analyses indicate that the majority of the changes measured in the 9 lipids profiled here occurred in the brainstem, hippocampus, midbrain, and striatum. To further illustrate this finding, Table 7 combines these analyses and shows the percent increase and decrease when comparing the chamber exposed to home cage control (Table 7(a)), chamber exposed to paced mated (Table 7(b)), and standard mated to paced mated (Table 7(c)). These data potentially represent a shift from appetitive neurochemistry (chamber exposed) to consummatory (paced mated) and indicate the brainstem, hippocampus, midbrain, and striatum as primary brain regions involved in this shift. Described below are the specific findings for each of these brain regions.

### 3.1. Brainstem Lipids across Treatment Groups

Brainstem levels of lipids showed the most dramatic differences from the home cage controls compared to the paced mated treatment group. Furthermore, 2-AG and PGE_2_ significantly increased in the chamber exposed group, whereas there was a significant decrease in SEA and 2-AG in the standard mated group (Table 3(a)). Levels of AEA, NAGly, PEA, SEA, OEA, DHEA, and 2-AG significantly decreased, whereas there was a significant increase in PGE_2_ in the paced mated group compared to home cage group (Table 3(a)). Likewise, there was a significant decrease in AEA, NAGly, PEA, SEA, OEA, and DHEA in the paced mated group compared to the chamber exposed group (Table 5(b)), as well as a significant decrease in AEA and 2-AG in the paced mated group compared to the standard mated group (Table 3(c)).

### 3.2. Hippocampus Lipid across Treatment Groups

Levels of NAGly, PEA, SEA, OEA, DHEA, and PGE2 showed significant increases in the chamber exposed group compared to the home cage group (Table 4(a)), whereas there were significant decreases in NAGly, PEA, SEA, OEA, and DHEA in the standard mated group, as well as significant decreases in AEA, NAGly, PEA, SEA, OEA, DHEA, PGE2, and PGF2*α* in the paced mated group compared to the chamber exposed group (Table 4(b)). In addition, there was a significant decrease in PEA, OEA, and DHEA in the paced mated group compared to the standard mated group (Table 4(c)).

### 3.3. Midbrain Lipids across Treatment Groups

Midbrain levels of the eCBs showed a unique profile in that there was a significant increase in AEA and a significant decrease in 2-AG in the chamber exposed group compared to the home cage group. Similarly, there was a significant decrease in 2-AG in the standard mated group compared to the home cage control (Table 5(a)). Comparisons of the chamber exposed group to the mating groups showed a significant decrease in AEA, NAGly, PEA, SEA, OEA, and DHEA in the paced mated group compared to the chamber exposed group (Table 5(b)). Likewise, there was a significant decrease in AEA, NAGly, PEA, SEA, OEA, and DHEA in the paced mated group compared to the standard mated group (Table 5(c)).

### 3.4. Striatum Lipids across Treatment Groups

Significant increases in PEA, OEA, DHEA, and 2-AG were demonstrated in the chamber exposed group compared to the home cage group. Additionally, there was a significant increase in SEA, PGE2, and PGF2*α* in the standard mated group compared to the home cage control (Table 6(a)). In comparison to chamber exposed controls, there were significant increases in PGE2 and PGF2*α* in the standard mated group and significant decreases in AEA, PEA, OEA, and DHEA in the paced mated group (Table 6(b)). Uniquely, every lipid measured was significantly lower in the paced mated group compared to the standard mated with the exception of the AEA metabolite, NAGly, which was significantly higher (Table 6(c)).

## 4. Discussion

### 4.1. Our Hypothesis That Lipid Analytes Would Be Altered by Mating Was Supported

Lipidomics, as a field, aims to identify and characterize biologically active lipids and their functional relevance. Here we have used lipidomics techniques to profile 9 signaling lipids throughout the female rat brain as a function of nonmating (chamber exposed and home cage controls) and mating strategies (standard verses paced). Each of the 9 lipids profiled here underwent differential regulation in at least one brain region in relation to the other treatment groups. The most consistent patterns of change were in those analytes measured in the brainstem, hippocampus, midbrain, and striatum.

### 4.2. Increases in Signaling Lipid Production in the Chambered Exposed Condition

There are at least two ways to interpret the overall increases in lipid signaling molecules during the chamber exposed condition: (1) as the neurochemical response to novelty stress (simply being moved to a new environment) or (2) as a neurochemical correlate to appetitive behavior (each of these rats had been tested for lordosis behavior with a male in this type of chamber 4 hours prior to the experimental treatment and may already associate the chamber with mating), though a combination of both scenarios may also be the case. Data shown here from the midbrain contributes to the theory that the chamber exposed group may be experiencing stress; previous work has shown that midbrain produces both AEA and 2-AG after the onset of stress [17]. Here, the midbrain showed an increase in AEA and a decrease in 2-AG in the chamber exposed group, which is somewhat at odds with the data by Hohmann and colleagues, in which 2-AG levels increase with stress [17]. Their time course for eCB measurement was much longer than the 15 minutes assayed here; therefore, the 15-minute period may have been too brief to show an increase in 2-AG. Alternatively, it could be an indication that the chamber exposed condition is a different type of stressor, for example, one with an ethological relationship to potential mating.

### 4.3. Decreases in Signaling Lipids in the Paced Mated Condition

Pacing behaviors among female rats may have deep evolutionary roots. For example, colonies of female rats live together in burrows that typically have entryways that are large enough only for an average-size female, but not an average-size male, to traverse. This enables females to leave their burrows and seek mating opportunities and retreat from males during mating, which allows them to control copulatory contacts [18, 19]. Both wild and laboratory bred rats exhibit these initiation and retreat patterns [19]. Therefore, female rats are able to control the type and number of sexual contacts and the intervals between them using a paced mating strategy. Paced mating, which is rewarding for female rats, facilitates hormonal release that is necessary to enter into an optimal progestational state. Estrogen and progesterone play a role in this feedback loop, as does prolactin, which is required for progestational uterine physiology and is particularly sensitive to paced mating versus standard mating.

Progesterone concentrations have a direct effect on how often a female will remove herself from contact with a male and for how long she stays away (higher progesterone typically means more contact with a male) [20]. One hypothesis to explain this phenomenon is that progesterone can act as an analgesic in genital sensitivity, given that female pacing can be influenced by pelvic nerve modulation [21]. Progesterone may also act as an anxiolytic, which may permit the female to withstand longer intromission time with males [22]. Also, estrogen in the striatum influences levels of dopamine and dopamine-mediated behaviors [23]. Dopamine, a neurohormone, at elevated concentrations within the nucleus accumbens (NAc) of the striatum, increases the percent of exits exhibited by female rats, suggesting that it may play a role in the level of neuronal feedback necessary to maintain longer reproductive bouts [23]. It has been predicted that once the level of dopamine begins to decline back to a basal state during mating, the females will return to copulate until the dopamine levels have been restored [23]. Of these two steroids, progesterone was found to have a stronger influence on pacing behavior [20]; however, some effects of progesterone to facilitate, and be increased by, sexual responding may occur through its actions as a prohormone. Progesterone is readily metabolized by sequential actions of 5*α*-reductase and 3*α*-hydroxysteroid oxidoreductase to form 5*α*-pregnan-3*α*-ol-20-one (3*α*,5*α*-THP), which is important in the production of paced mating [13]. 3*α*,5*α*-THP, unlike progesterone, does not act on progestin receptors (PRs), has actions via GABA_A_, NMDA, dopamine receptors, and downstream signal transduction pathways, which may contribute to the reward state associated with paced mating [13].

Diurnal prolactin secretion following mating is more readily instantiated among rats which receive ten or more intromissions during a paced copulatory series as compared to five or fewer sexual contacts in the control, standard mating, paradigm [24]. Controlling the number of intromissions and intervals between them, in paced mating, results in more litters coming to terms and larger litter sizes than what occurs with the standard mating procedure [25]. Enhanced fertility and fecundity with paced, compared to standard, mating may be a result of neurophysiological changes in the brain and reproductive tract [24]. Indeed, female rats engaged in paced mating have earlier termination of estrus [22] and begin twice daily prolactin surges, characteristic of pregnancy, and have higher levels of progestogens, sooner, than do their nonpaced counterparts [20, 25]. This optimal hormonal stimulation in paced mated female rats may facilitate reward processes and increases the probability of subsequent pregnancies [19, 26].

Rewarding properties of many psychoactive drugs are initiated by activation of the mesolimbic dopaminergic system that begins in the ventral tegmental area of the midbrain (VTA) [27]. Our understanding of how the cannabinoid system plays a role in reward circuitry is growing but still poorly characterized [28]. Increases in AEA and 2-AG production were demonstrated in the midbrain when rats experience chronic alcohol exposure [29], whereas 2-AG and AEA decreased in animals exposed to chronic amphetamine [30]. Both of these results have implications for interactions with the midbrain-striatal dopamine system. Paced mating strategies result in higher levels of midbrain dopamine than standard mating, and this appears to be regulated by estrogen acting on striatal neurons [23]. Here, we show that midbrain AEA and striatal AEA and 2-AG levels are significantly lower after 15 minutes of paced mating compared to standard mating. These data suggest that the paced mating paradigm could be a model for studying the interactions between the cannabinoid and dopaminergic systems in the context of acute rewards without using exogenous pharmacological interventions.

### 4.4. *N*-Acylethanolamines Show Dramatic Differences in Regulation in Brainstem, Hippocampus, Midbrain, and Striatum

*N*-Acylethanolamines are a large family of lipid signaling molecules that are ubiquitous in nature. They are primary signaling molecules in plants [31], invertebrates [32], and vertebrates [6]. All of the signaling properties of each of these lipids are not fully understood. PEA, OEA, and DHEA have been shown to have “cannabimimetic” properties in which they are associated with anti-inflammatory and analgesic responses [33]. Recently, OEA was proposed to be the endogenous ligand for GPR119 [34], whereas PEA is an activator of P-PAR-alpha [35]. In our hands, DHEA activates TRPV1 receptors with the same efficacy as AEA (*unpublished results*); however, to date there is no specific molecular target for SEA. Data here show that the metabolism and production of this family of signaling lipids are most acutely changed in female rats that are either placed in the mating area (chamber exposed) or paced mated. Fatty acid amide hydrolase (FAAH) is the primary regulatory enzyme for the family of *N*-acylethanolamine molecules [36]. Recent work from our lab also suggests that FAAH is the rate-limiting factor for NAGly biosynthesis [7]. Earlier evidence suggested that FAAH has an estrogen response element in its promoter region [5]. Therefore, estrogen priming occurring concurrent to ovulation may prime the system to have more FAAH available for rapid degradation of *N*-acyl amides. The rapidity in which these *N*-acylethanolamines appear to first increase in production upon introduction to the mating arena and then rapidly degrade in the context of paced mating suggests a more flexible and timely system than one that requires gene transcription. The wide range of molecular targets of the *N*-acylethanolamines measured here also suggests a variety of regulatory mechanisms that are likely activated with these dramatic changes in signaling ligands.

Overall changes in the majority of lipids profiled here were concentrated in the midbrain, striatum, and hippocampus. Our prior work has demonstrated that other brain neurotrophic factors, neurosteroids, are also changed with mating [26, 37–39]. Indeed, we have consistently seen and reported that levels of allopregnanolone in the midbrain VTA (but also the hippocampus > striatum, cortex) increase with mating these effects are greater with paced mating than; with standard mating, and they occur among gonadally intact rats or rats that are ovariectomized and adrenalectomized and estrogen-primed. Our work on this topic has suggested that estrogen enhances biosynthesis of neurosteroids, which may help prime females and enable them to seek out and/or initiate mating. However, with mating there is a further rise in steroid biosynthesis and then a decline which is necessary for termination of mating (and reproductive success). Interestingly, here we see that other lipid signaling molecules are also labile and changing in response to acute reproductive experience.

## 5. Conclusions

Neurochemical regulation of complex behavioral patterns, such as the appetitive and consummatory aspects of mating behaviors in females, requires hormonal priming but is rapidly modified within the mating context. Here, we add to this intricate neurophysiological signaling event by demonstrating that eCBs, PGs, and the *N*-acylethanolamines PEA, SEA, OEA, and DHEA are, likewise, modified during these situations. Brain areas that predominate in reward system pathways that are engaged during paced mating show the most dramatic changes in these lipid signaling molecules suggesting a role for these lipids in how this reward system is both activated and maintained. We hope to bear out the functional significance of these changes in our future work.

## Figures and Tables

**Table 1:** General linear model analyses of each lipid profiled by brain area and treatment group.

GLM analysis								
	BS	CER	HIPP	HYP	MB	OB	STR	THAL
AEA	**0.008**	0.077	0.093	0.319	**0.025**	0.819	**0.017**	0.070
NAGly	**0.037**	0.069	**0.039**	0.224	0.076	0.802	0.241	0.128
PEA	**0.009**	0.119	**0.001**	0.331	**0.039**	0.528	**0.017**	0.143
SEA	**0.016**	0.103	**0.000**	0.665	0.065	0.785	**0.039**	0.589
OEA	**0.017**	0.103	**0.000**	0.588	**0.028**	0.171	**0.012**	0.114
DHEA	**0.042**	0.056	**0.001**	0.284	**0.035**	0.567	**0.009**	0.181
2-AG	**0.000**	0.332	0.572	0.975	**0.020**	0.465	**0.007**	0.224
PGE2	**0.047**	0.283	**0.046**	0.187	0.348	0.597	**0.000**	0.979
PGF2*α*	0.419	0.113	0.079	0.168	0.306	0.731	**0.000**	0.656

*P* ≤ 0.05. Significant differences are shown in bold black.

**Table 2:** Levels of lipid production in olfactory bulbs, hypothalamus, thalamus, and cerebellum across the four treatment groups: home cage control, chamber exposed, standard mated, and paced mated.

	Home cage	Chamber exposed	Standard mated	Paced mated
Olfactory bulbs				
AEA	8.2*E* − 11 ± 2.4*E* − 11	6.8*E* − 11 ± 1.0*E* − 11	7.1*E* − 11 ± 1.6*E* − 11	9.8*E* − 11 ± 3.8*E* − 11
NAGly	1.8*E* − 11 ± 5.4*E* − 12	2.3*E* − 11 ± 4.4*E* − 12	1.7*E* − 11 ± 4.9*E* − 12	3.1*E* − 11 ± 2.0*E* − 11
PEA	4.1*E* − 12 ± 1.2*E* − 12	3.9*E* − 12 ± 9.6*E* − 13	3.7*E* − 12 ± 1.3*E* − 12	2.2*E* − 12 ± 3.0*E* − 13
SEA	6.2*E* − 11 ± 1.5*E* − 11	6.4*E* − 11 ± 1.5*E* − 11	6.0*E* − 11 ± 1.8*E* − 11	4.5*E* − 11 ± 6.9*E* − 12
OEA	8.4*E* − 11 ± 1.6*E* − 11	9.3*E* − 11 ± 1.3*E* − 11	8.2*E* − 11 ± 2.1*E* − 11	4.7*E* − 11 ± 4.3*E* − 12
DHEA	4.3*E* − 11 ± 1.5*E* − 11	2.7*E* − 11 ± 3.1*E* − 12	5.6*E* − 11 ± 2.1*E* − 11	3.5*E* − 11 ± 1.4*E* − 11
2-AG	2.6*E* − 9 ± 1.3*E* − 10	2.1*E* − 9 ± 2.9*E* − 10	2.6*E* − 9 ± 3.2*E* − 10	2.6*E* − 9 ± 2.7*E* − 10
PGE2	9.1*E* − 10 ± 1.6*E* − 10	8.1*E* − 10 ± 1.9*E* − 10	6.5*E* − 10 ± 1.8*E* − 10	6.5*E* − 10 ± 9.4*E* − 11
PGF2*α*	3.0*E* − 10 ± 5.3*E* − 11	2.5*E* − 10 ± 5.5*E* − 11	2.1*E* − 10 ± 5.2*E* − 11	3.2*E* − 10 ± 1.1*E* − 10

Hypothalamus				
AEA	1.2*E* − 10 ± 8.0*E* − 11	1.4*E* − 11 ± 1.9*E* − 11	9.8*E* − 11 ± 6.6*E* − 12	1.1*E* − 10 ± 2.3*E* − 11
NAGly	7.1*E* − 12 ± 1.6*E* − 12	1.1*E* − 11 ± 2.2*E* − 12	6.1*E* − 12 ± 1.5*E* − 12	1.0*E* − 11 ± 2.0*E* − 12
PEA	1.4*E* − 10 ± 1.9*E* − 11	1.0*E* − 11 ± 1.3*E* − 12	7.1*E* − 12 ± 5.2*E* − 13	9.0*E* − 12 ± 3.1*E* − 12
SEA	1.8*E* − 10 ± 2.5*E* − 11	1.7*E* − 10 ± 2.3*E* − 11	1.5*E* − 10 ± 1.6*E* − 11	2.1*E* − 10 ± 7.8*E* − 11
OEA	1.6*E* − 11 ± 2.2*E* − 12	2.6*E* − 10 ± 2.7*E* − 11	1.9*E* − 10 ± 2.2*E* − 11	2.5*E* − 10 ± 8.9*E* − 11
DHEA	7.6*E* − 9 ± 1.0*E* − 9	3.0*E* − 11 ± 8.9*E* − 12	2.4*E* − 11 ± 3.0*E* − 12	3.0*E* − 11 ± 10.0*E* − 12
2-AG	7.6*E* − 9 ± 1.0*E* − 9	8.5*E* − 9 ± 3.0*E* − 9	7.4*E* − 9 ± 2.3*E* − 9	7.2*E* − 9 ± 1.6*E* − 9
PGE2	2.4*E* − 10 ± 7.1*E* − 11	4.0*E* − 10 ± 7.5*E* − 11	2.9*E* − 10 ± 5.9*E* − 11	5.0*E* − 10 ± 1.3*E* − 10
PGF2*α*	1.6*E* − 10 ± 3.0*E* − 11	2.1*E* − 10 ± 2.4*E* − 11	1.7*E* − 10 ± 3.0*E* − 11	2.7*E* − 10 ± 5.5*E* − 11

Thalamus				
AEA	3.3*E* − 11 ± 1.5*E* − 12	3.7*E* − 11 ± 6.8*E* − 12	3.7*E* − 11 ± 4.9*E* − 12	2.2*E* − 11 ± 1.7*E* − 12
NAGly	1.6*E* − 11 ± 2.1*E* − 12	2.0*E* − 11 ± 3.6*E* − 12	1.9*E* − 11 ± 2.2*E* − 12	1.2*E* − 11 ± 5.9*E* − 13
PEA	2.4*E* − 11 ± 1.8*E* − 12	3.1*E* − 11 ± 6.2*E* − 12	2.6*E* − 11 ± 3.8*E* − 12	1.8*E* − 11 ± 1.1*E* − 12
SEA	3.5*E* − 10 ± 2.8*E* − 11	4.2*E* − 10 ± 7.0*E* − 11	3.9*E* − 10 ± 3.5*E* − 11	3.5*E* − 10 ± 2.7*E* − 11
OEA	4.9*E* − 10 ± 3.4*E* − 11	6.2*E* − 10 ± 1.1*E* − 10	5.6*E* − 10 ± 6.7*E* − 11	3.8*E* − 10 ± 2.9*E* − 11
DHEA	1.1*E* − 10 ± 5.7*E* − 12	1.4*E* − 10 ± 1.8*E* − 11	1.3*E* − 10 ± 1.3*E* − 11	1.0*E* − 10 ± 6.5*E* − 12
2-AG	1.8*E* − 8 ± 1.4*E* − 9	1.5*E* − 8 ± 10.0*E* − 10	1.8*E* − 8 ± 1.0*E* − 9	1.8*E* − 8 ± 1.2*E* − 9
PGE2	1.8*E* − 10 ± 2.3*E* − 11	1.9*E* − 10 ± 4.6*E* − 11	2.0*E* − 10 ± 5.1*E* − 11	1.9*E* − 10 ± 2.0*E* − 11
PGF2*α*	2.2*E* − 10 ± 1.9*E* − 11	1.8*E* − 10 ± 2.7*E* − 11	2.0*E* − 10 ± 3.0*E* − 11	1.8*E* − 10 ± 1.5*E* − 11

Cerebellum				
AEA	3.4*E* − 12 ± 4.4*E* − 13	5.4*E* − 12 ± 1.1*E* − 12	3.2*E* − 12 ± 2.4*E* − 13	4.0*E* − 12 ± 2.6*E* − 13
NAGly	7.3*E* − 12 ± 3.3*E* − 13	9.3*E* − 12 ± 1.5*E* − 12	7.7*E* − 12 ± 4.7*E* − 13	5.9*E* − 12 ± 4.2*E* − 13
PEA	4.4*E* − 12 ± 2.5*E* − 13	6.6*E* − 12 ± 1.2*E* − 12	4.8*E* − 12 ± 1.7*E* − 13	4.5*E* − 12 ± 5.6*E* − 13
SEA	9.5*E* − 11 ± 5.4*E* − 12	1.4*E* − 10 ± 2.1*E* − 11	1.2*E* − 10 ± 3.5*E* − 12	1.1*E* − 10 ± 1.1*E* − 11
OEA	1.1*E* − 10 ± 3.9*E* − 12	1.6*E* − 10 ± 3.0*E* − 11	1.2*E* − 10 ± 4.9*E* − 12	1.1*E* − 10 ± 9.6*E* − 12
DHEA	2.1*E* − 11 ± 7.0*E* − 13	3.1*E* − 11 ± 4.9*E* − 12	2.3*E* − 11 ± 2.7*E* − 13	2.6*E* − 11 ± 1.9*E* − 12
2-AG	3.2*E* − 9 ± 1.6*E* − 10	3.4*E* − 9 ± 1.7*E* − 10	3.3*E* − 9 ± 1.3*E* − 10	3.9*E* − 9 ± 4.5*E* − 10
PGE2	9.5*E* − 11 ± 1.1*E* − 11	8.4*E* − 11 ± 8.3*E* − 12	1.1*E* − 10 ± 9.2*E* − 12	8.0*E* − 11 ± 1.7*E* − 11
PGF2*α*	5.3*E* − 11 ± 4.8*E* − 12	5.1*E* − 11 ± 8.3*E* − 12	5.6*E* − 11 ± 3.6*E* − 12	3.7*E* − 11 ± 4.4*E* − 12

**Table:** (a) Comparisons of home cage to each group

Brainstem: significance versus home cage control				
	Home cage	Chamber exposed	Standard mated	Paced mated
AEA	1.7*E* − 11 ± 1.7*E* − 12	2.0*E* − 11 ± 2.5*E* − 12	1.6*E* − 11 ± 2.1*E* − 12	9.6***E*** − 12 ± 9.8***E*** − 13
NAGly	2.1*E* − 11 ± 8.4*E* − 13	2.3*E* − 11 ± 3.0*E* − 12	2.0*E* − 11 ± 1.1*E* − 12	1.5***E*** − 11 ± 1.8***E*** − 12

PEA	3.4*E* − 11 ± 1.2*E* − 12	3.9*E* − 11 ± 4.1*E* − 12	3.0*E* − 12 ± 3.7*E* − 12	2.3***E*** − 11 ± 2.2***E*** − 12
SEA	6.5*E* − 10 ± 4.5*E* − 11	5.8*E* − 10 ± 5.4*E* − 11	5.2***E*** − 10 ± 3.0***E*** − 11	4.5***E*** − 10 ± 3.6***E*** − 11
OEA	8.3*E* − 10 ± 3.2*E* − 11	8.9*E* − 10 ± 1.1*E* − 10	7.3*E* − 10 ± 6.3*E* − 11	5.4***E*** − 10 ± 7.1***E*** − 11
DHEA	1.3*E* − 10 ± 4.6*E* − 12	1.3*E* − 10 ± 1.5*E* − 11	1.2*E* − 10 ± 7.6*E* − 12	9.3***E*** − 11 ± 1.2***E*** − 11

2-AG	2.1*E* − 8 ± 6.9*E* − 10	1.4*E* − 8 ± 1.1*E* − 9^†^	1.6***E*** − 8 ± 7.4***E*** − 10	1.2***E*** − 8 ± 8.1***E*** − 10

PGE2	2.1*E* − 10 ± 2.6*E* − 11	3.4*E* − 10 ± 2.8*E* − 11^†^	2.8*E* − 10 ± 3.5*E* − 11	3.3*E* − 10 ± 4.0*E* − 11^†^
PGF2*α*	1.7*E* − 10 ± 1.0*E* − 11	2.0*E* − 10 ± 1.5*E* − 11	1.8*E* − 10 ± 6.8*E* − 12	1.6*E* − 10 ± 2.2*E* − 11

**Table:** (b) Comparisons of chamber exposed to standard or paced mating

Brainstem: standard and paced mating versus chamber exposed			
	Chamber exposed	Standard mated	Paced mated
AEA	2.0*E* − 11 ± 2.5*E* − 12	1.6*E* − 11 ± 2.1*E* − 12	9.6***E*** − 12 ± 9.8***E*** − 13
NAGly	2.3*E* − 11 ± 3.0*E* − 12	2.0*E* − 11 ± 1.1*E* − 12	1.5***E*** − 11 ± 1.8***E*** − 12

PEA	3.9*E* − 11 ± 4.1*E* − 12	3.0*E* − 12 ± 3.7*E* − 12	2.3***E*** − 11 ± 2.2***E*** − 12
SEA	5.8*E* − 10 ± 5.4*E* − 11	5.2*E* − 10 ± 3.0*E* − 11	4.5***E*** − 10 ± 3.6***E*** − 11
OEA	8.9*E* − 10 ± 1.1*E* − 10	7.3*E* − 10 ± 6.3*E* − 11	5.4***E*** − 10 ± 7.1***E*** − 11
DHEA	1.3*E* − 10 ± 1.5*E* − 11	1.2*E* − 10 ± 7.6*E* − 12	9.3***E*** − 11 ± 1.2***E*** − 11

2-AG	1.4*E* − 8 ± 1.1*E* − 9	1.6*E* − 8 ± 7.4*E* − 10	1.2*E* − 8 ± 8.1*E* − 10

PGE2	3.4*E* − 10 ± 2.8*E* − 11	2.8*E* − 10 ± 3.5*E* − 11	3.3*E* − 10 ± 4.0*E* − 11
PGF2*α*	2.0*E* − 10 ± 1.5*E* − 11	1.8*E* − 10 ± 6.8*E* − 12	1.6*E* − 10 ± 2.2*E* − 11

**Table:** (c) Comparisons of standard mating to paced mating

Brainstem: standard versus paced mating		
	Standard mated	Paced mated
AEA	1.6*E* − 11 ± 2.1*E* − 12	9.6***E*** − 12 ± 9.8***E*** − 13
NAGly	2.0*E* − 11 ± 1.1*E* − 12	1.5*E* − 11 ± 1.8*E* − 12

PEA	3.0*E* − 12 ± 3.7*E* − 12	2.3*E* − 11 ± 2.2*E* − 12
SEA	5.2*E* − 10 ± 3.0*E* − 11	4.5*E* − 10 ± 3.6*E* − 11
OEA	7.3*E* − 10 ± 6.3*E* − 11	5.4*E* − 10 ± 7.1*E* − 11
DHEA	1.2*E* − 10 ± 7.6*E* − 12	9.3*E* − 11 ± 1.2*E* − 11

2-AG	1.6*E* − 8 ± 7.4*E* − 10	1.2***E*** − 8 ± 8.1***E*** − 10

PGE2	2.8*E* − 10 ± 3.5*E* − 11	3.3*E* − 10 ± 4.0*E* − 11
PGF2*α*	1.8*E* − 10 ± 6.8*E* − 12	1.6*E* − 10 ± 2.2*E* − 11

Data are moles per gram tissue and are shown as means ± SE. Values in light face have no significant difference among the groups. ^†^Values denote a significant increase, whereas those in bold denote a significant decrease from the treatment group in the far left. *P* ≤ 0.05.

**Table:** (a) Comparisons of home cage to each group

Hippocampus: significance versus home cage control				
	Home cage	Chamber exposed	Standard mated	Paced mated
AEA	6.3*E* − 11 ± 7.1*E* − 12	7.0*E* − 11 ± 1.5*E* − 11	5.2*E* − 11 ± 8.2*E* − 12	3.6*E* − 11 ± 3.7*E* − 12
NAGly	2.3*E* − 11 ± 1.6*E* − 12	4.2*E* − 11 ± 1.1*E* − 11^†^	2.5*E* − 11 ± 2.1*E* − 12	1.8*E* − 11 ± 1.4*E* − 12

PEA	6.7*E* − 12 ± 6.2*E* − 13	1.4*E* − 11 ± 2.2*E* − 12^†^	9.0*E* − 12 ± 1.1*E* − 12	5.0*E* − 12 ± 3.9*E* − 13
SEA	1.2*E* − 10 ± 1.3*E* − 11	2.4*E* − 10 ± 2.9*E* − 11^†^	1.5*E* − 10 ± 1.9*E* − 11	1.2*E* − 10 ± 1.1*E* − 11
OEA	2.1*E* − 10 ± 1.8*E* − 11	3.2*E* − 10 ± 3.3*E* − 11^†^	2.5*E* − 10 ± 2.7*E* − 11	1.5*E* − 10 ± 6.5*E* − 12
DHEA	4.2*E* − 11 ± 3.9*E* − 12	6.8*E* − 11 ± 5.8*E* − 12^†^	5.0*E* − 11 ± 6.3*E* − 12	3.4*E* − 11 ± 1.6*E* − 12

2-AG	9.2*E* − 9 ± 4.5*E* − 10	8.7*E* − 9 ± 1.4*E* − 9	7.4*E* − 9 ± 1.0*E* − 9	8.0*E* − 9 ± 5.9*E* − 10

PGE2	2.7*E* − 10 ± 4.7*E* − 11	5.9*E* − 10 ± 1.7*E* − 10^†^	3.4*E* − 10 ± 4.7*E* − 11	2.2*E* − 10 ± 2.9*E* − 11
PGF2*α*	2.9*E* − 10 ± 2.4*E* − 11	3.9*E* − 10 ± 8.8*E* − 11	3.0*E* − 10 ± 3.1*E* − 11	2.0*E* − 10 ± 2.1*E* − 11

**Table:** (b) Comparisons of chamber exposed to standard or paced mating

Hippocampus: standard and paced mating versus chamber exposed			
	Chamber exposed	Standard mated	Paced mated
AEA	7.0*E* − 11 ± 1.5*E* − 11	5.2*E* − 11 ± 8.2*E* − 12	3.6***E*** − 11 ± 3.7***E*** − 12
NAGly	4.2*E* − 11 ± 1.1*E* − 11	2.5***E*** − 11 ± 2.1***E*** − 12	1.8***E*** − 11 ± 1.4***E*** − 12

PEA	1.4*E* − 11 ± 2.2*E* − 12	9.0***E*** − 12 ± 1.1***E*** − 12	5.0***E*** − 12 ± 3.9***E*** − 13
SEA	2.4*E* − 10 ± 2.9*E* − 11	1.5***E*** − 10 ± 1.9***E*** − 11	1.2***E*** − 10 ± 1.1***E*** − 11
OEA	3.2*E* − 10 ± 3.3*E* − 11	2.5***E*** − 10 ± 2.7***E*** − 11	1.5***E*** − 10 ± 6.5***E*** − 12
DHEA	6.8*E* − 11 ± 5.8*E* − 12	5.0***E*** − 11 ± 6.3***E*** − 12	3.4***E*** − 11 ± 1.6***E*** − 12

2-AG	8.7*E* − 9 ± 1.4*E* − 9	7.4*E* − 9 ± 1.0*E* − 9	8.0*E* − 9 ± 5.9*E* − 10

PGE2	5.9*E* − 10 ± 1.7*E* − 10	3.4*E* − 10 ± 4.7*E* − 11	2.2***E*** − 10 ± 2.9***E*** − 11
PGF2*α*	3.9*E* − 10 ± 8.8*E* − 11	3.0*E* − 10 ± 3.1*E* − 11	2.0***E*** − 10 ± 2.1***E*** − 11

**Table:** (c) Comparisons of standard mating to paced mating

Hippocampus: standard versus paced mating		
	Standard mated	Paced mated
AEA	5.2*E* − 11 ± 8.2*E* − 12	3.6*E* − 11 ± 3.7*E* − 12
NAGly	2.5*E* − 11 ± 2.1*E* − 12	1.8*E* − 11 ± 1.4*E* − 12

PEA	9.0*E* − 12 ± 1.1*E* − 12	5.0***E*** − 12 ± 3.9***E*** − 13
SEA	1.5*E* − 10 ± 1.9*E* − 11	1.2*E* − 10 ± 1.1*E* − 11
OEA	2.5*E* − 10 ± 2.7*E* − 11	1.5***E*** − 10 ± 6.5***E*** − 12
DHEA	5.0*E* − 11 ± 6.3*E* − 12	3.4***E*** − 11 ± 1.6***E*** − 12

2-AG	7.4*E* − 9 ± 1.0*E* − 9	8.0*E* − 9 ± 5.9*E* − 10

PGE2	3.4*E* − 10 ± 4.7*E* − 11	2.2*E* − 10 ± 2.9*E* − 11
PGF2*α*	3.0*E* − 10 ± 3.1*E* − 11	2.0*E* − 10 ± 2.1*E* − 11

Data are moles per gram tissue and are shown as means ± SE. Values in light face have no significant difference among the groups. ^†^Values denote a significant increase, whereas those in bold denote a significant decrease from the treatment group in the far left. *P* ≤ 0.05.

**Table:** (a) Comparisons of home cage to each group

Midbrain: significance versus home cage control				
	Home cage	Chamber exposed	Standard mated	Paced mated
AEA	1.2*E* − 11 ± 1.1*E* − 12	1.8*E* − 11 ± 2.9*E* − 12^†^	1.5*E* − 11 ± 1.3*E* − 12	1.0*E* − 11 ± 7.3*E* − 13
NAGly	9.7*E* − 12 ± 1.1*E* − 12	1.3*E* − 11 ± 2.2*E* − 12	9.4*E* − 12 ± 7.1*E* − 13	7.8*E* − 12 ± 2.7*E* − 13

PEA	8.8*E* − 12 ± 6.2*E* − 13	1.2*E* − 11 ± 2.1*E* − 12	1.1*E* − 11 ± 9.4*E* − 13	7.3*E* − 12 ± 4.5*E* − 13
SEA	1.6*E* − 10 ± 6.8*E* − 12	2.0*E* − 10 ± 2.6*E* − 11	2.1*E* − 10 ± 1.7*E* − 11	1.5*E* − 10 ± 10.0*E* − 12
OEA	2.2*E* − 10 ± 1.5*E* − 11	3.1*E* − 10 ± 5.4*E* − 11	2.9*E* − 10 ± 2.5*E* − 11	1.8*E* − 10 ± 1.2*E* − 11
DHEA	5.3*E* − 11 ± 3.1*E* − 12	6.9*E* − 11 ± 9.6*E* − 12	6.8*E* − 11 ± 4.2*E* − 12	4.7*E* − 11 ± 3.3*E* − 12

2-AG	1.3*E* − 8 ± 6.8*E* − 10	9.7***E*** − 9 ± 7.9***E*** − 10	1.1***E*** − 8 ± 6.4***E*** − 10	1.1*E* − 8 ± 4.0*E* − 10

PGE2	3.7*E* − 10 ± 5.6*E* − 11	1.1*E* − 10 ± 2.2*E* − 11	2.4*E* − 10 ± 2.5*E* − 11	2.7*E* − 10 ± 8.0*E* − 11
PGF2*α*	1.2*E* − 10 ± 1.6*E* − 11	1.3*E* − 11 ± 2.2*E* − 12	8.2*E* − 11 ± 9.0*E* − 12	8.7*E* − 11 ± 1.4*E* − 11

**Table:** (b) Comparisons of chamber exposed to standard or paced mating

Midbrain: standard and paced mating versus chamber exposed			
	Chamber exposed	Standard mated	Paced mated
AEA	1.8*E* − 11 ± 2.9*E* − 12	1.5*E* − 11 ± 1.3*E* − 12	1.0***E*** − 11 ± 7.3***E*** − 13
NAGly	1.3*E* − 11 ± 2.2*E* − 12	9.4*E* − 12 ± 7.1*E* − 13	7.8***E*** − 12 ± 2.7***E*** − 13

PEA	1.2*E* − 11 ± 2.1*E* − 12	1.1*E* − 11 ± 9.4*E* − 13	7.3***E*** − 12 ± 4.5***E*** − 13
SEA	2.0*E* − 10 ± 2.6*E* − 11	2.1*E* − 10 ± 1.7*E* − 11	1.5***E*** − 10 ± 10.0***E*** − 12
OEA	3.1*E* − 10 ± 5.4*E* − 11	2.9*E* − 10 ± 2.5*E* − 11	1.8***E*** − 10 ± 1.2***E*** − 11
DHEA	6.9*E* − 11 ± 9.6*E* − 12	6.8*E* − 11 ± 4.2*E* − 12	4.7***E*** − 11 ± 3.3***E*** − 12

2-AG	9.7*E* − 9 ± 7.9*E* − 10	1.1*E* − 8 ± 6.4*E* − 10	1.1*E* − 8 ± 4.0*E* − 10

PGE2	1.1*E* − 10 ± 2.2*E* − 11	2.4*E* − 10 ± 2.5*E* − 11	2.7*E* − 10 ± 8.0*E* − 11
PGF2*α*	1.3*E* − 11 ± 2.2*E* − 12	8.2*E* − 11 ± 9.0*E* − 12	8.7*E* − 11 ± 1.4*E* − 11

**Table:** (c) Comparisons of standard mating to paced mating

Midbrain: standard versus paced mating		
	Standard mated	Paced mated
AEA	1.5*E* − 11 ± 1.3*E* − 12	1.0***E*** − 11 ± 7.3***E*** − 13
NAGly	9.4*E* − 12 ± 7.1*E* − 13	7.8***E*** − 12 ± 2.7***E*** − 13

PEA	1.1*E* − 11 ± 9.4*E* − 13	7.3***E*** − 12 ± 4.5***E*** − 13
SEA	2.1*E* − 10 ± 1.7*E* − 11	1.5***E*** − 10 ± 10.0***E*** − 12
OEA	2.9*E* − 10 ± 2.5*E* − 11	1.8***E*** − 10 ± 1.2***E*** − 11
DHEA	6.8*E* − 11 ± 4.2*E* − 12	4.7***E*** − 11 ± 3.3***E*** − 12

2-AG	1.1*E* − 8 ± 6.4*E* − 10	1.1*E* − 8 ± 4.0*E* − 10

PGE2	2.4*E* − 10 ± 2.5*E* − 11	2.7*E* − 10 ± 8.0*E* − 11
PGF2*α*	8.2*E* − 11 ± 9.0*E* − 12	8.7*E* − 11 ± 1.4*E* − 11

Data are moles per gram tissue and are shown as means ± SE. Values in light face have no significant difference among the groups. ^†^Values denote a significant increase, whereas those in bold denote a significant decrease from the treatment group in the far left. *P* ≤ 0.05.

**Table:** (a) Comparisons of home cage to each group

Striatum: significance versus home cage control				
	Home cage	Chamber exposed	Standard mated	Paced mated
AEA	2.3*E* − 12 ± 2.5*E* − 13	2.9*E* − 12 ± 3.4*E* − 13	3.1*E* − 12 ± 5.0*E* − 13	1.6*E* − 12 ± 7.3*E* − 14
NAGly	5.8*E* − 11 ± 1.4*E* − 11	7.0*E* − 11 ± 1.2*E* − 11	7.8*E* − 11 ± 1.2*E* − 11	9.6*E* − 11 ± 1.3*E* − 11

PEA	6.1*E* − 13 ± 5.3*E* − 14	1.0*E* − 12 ± 1.6*E* − 13^†^	8.8*E* − 13 ± 1.3*E* − 13	5.3*E* − 13 ± 2.1*E* − 14
SEA	8.4*E* − 12 ± 7.6*E* − 13	1.0*E* − 11 ± 1.2*E* − 12	1.2*E* − 11 ± 1.4*E* − 12^†^	7.2*E* − 12 ± 6.0*E* − 13
OEA	9.6*E* − 12 ± 8.1*E* − 13	1.5*E* − 11 ± 2.3*E* − 12^†^	1.4*E* − 11 ± 2.1*E* − 12	8.2*E* − 12 ± 2.6*E* − 13
DHEA	3.1*E* − 12 ± 2.7*E* − 13	4.4*E* − 12 ± 6.1*E* − 13^†^	4.0*E* − 12 ± 4.6*E* − 13	2.4*E* − 12 ± 2.0*E* − 13

2-AG	3.4*E* − 10 ± 1.3*E* − 11	4.1*E* − 10 ± 2.8*E* − 11^†^	3.8*E* − 10 ± 1.7*E* − 11	3.1*E* − 10 ± 1.5*E* − 11

PGE2	9.6*E* − 11 ± 1.5*E* − 11	1.2*E* − 10 ± 2.7*E* − 11	2.4*E* − 10 ± 2.5*E* − 11^†^	9.8*E* − 11 ± 1.5*E* − 11
PGF2*α*	1.0*E* − 10 ± 8.5*E* − 12	1.2*E* − 10 ± 1.6*E* − 11	1.8*E* − 10 ± 1.3*E* − 11^†^	8.7*E* − 11 ± 1.2*E* − 11

**Table:** (b) Comparisons of chamber exposed to standard or paced mating

Striatum: standard and paced mating versus chamber exposed			
	Chamber exposed	Standard mated	Paced mated
AEA	2.9*E* − 12 ± 3.4*E* − 13	3.1*E* − 12 ± 5.0*E* − 13	1.6***E*** − 12 ± 7.3***E*** − 14
NAGly	7.0*E* − 11 ± 1.2*E* − 11	7.8*E* − 11 ± 1.2*E* − 11	9.6*E* − 11 ± 1.3*E* − 11

PEA	1.0*E* − 12 ± 1.6*E* − 13	8.8*E* − 13 ± 1.3*E* − 13	5.3***E*** − 13 ± 2.1***E*** − 14
SEA	1.0*E* − 11 ± 1.2*E* − 12	1.2*E* − 11 ± 1.4*E* − 12	7.2*E* − 12 ± 6.0*E* − 13
OEA	1.5*E* − 11 ± 2.3*E* − 12	1.4*E* − 11 ± 2.1*E* − 12	8.2***E*** − 12 ± 2.6***E*** − 13
DHEA	4.4*E* − 12 ± 6.1*E* − 13	4.0*E* − 12 ± 4.6*E* − 13	2.4***E*** − 12 ± 2.0***E*** − 13

2-AG	4.1*E* − 10 ± 2.8*E* − 11	3.8*E* − 10 ± 1.7*E* − 11	3.1***E*** − 10 ± 1.5***E*** − 11

PGE2	1.2*E* − 10 ± 2.7*E* − 11	2.4*E* − 10 ± 2.5*E* − 11^†^	9.8*E* − 11 ± 1.5*E* − 11
PGF2*α*	1.2*E* − 10 ± 1.6*E* − 11	1.8*E* − 10 ± 1.3*E* − 11^†^	8.7*E* − 11 ± 1.2*E* − 11

**Table:** (c) Comparisons of standard mating to paced mating

Striatum: standard versus paced mating		
	Standard mated	Paced mated
AEA	3.1*E* − 12 ± 5.0*E* − 13	1.6***E*** − 12 ± 7.3***E*** − 14
NAGly	7.8*E* − 11 ± 1.2*E* − 11	9.6*E* − 11 ± 1.3*E* − 11^†^

PEA	8.8*E* − 13 ± 1.3*E* − 13	5.3***E*** − 13 ± 2.1***E*** − 14
SEA	1.2*E* − 11 ± 1.4*E* − 12	7.2***E*** − 12 ± 6.0***E*** − 13
OEA	1.4*E* − 11 ± 2.1*E* − 12	8.2***E*** − 12 ± 2.6***E*** − 13
DHEA	4.0*E* − 12 ± 4.6*E* − 13	2.4***E*** − 12 ± 2.0***E*** − 13

2-AG	3.8*E* − 10 ± 1.7*E* − 11	3.1***E*** − 10 ± 1.5***E*** − 11

PGE2	2.4*E* − 10 ± 2.5*E* − 11	9.8***E*** − 11 ± 1.5***E*** − 11
PGF2*α*	1.8*E* − 10 ± 1.3*E* − 11	8.7***E*** − 11 ± 1.2***E*** − 11

Data are moles per gram tissue and are shown as means ± SE. Values in light face have no significant difference among the groups. ^†^Values denote a significant increase, whereas those in bold denote a significant decrease from the treatment group in the far left. *P* ≤ 0.05.

**Table:** (a)

Chamber exposed compared to home cage control				
	Hippocampus	Midbrain	Striatum	Brainstem
AEA		↑(50%)		
NAGly	↑(83%)			
PEA	↑(109%)		↑(64%)	
SEA	↑(100%)			
OEA	↑(52%)		↑(56%)	
DHEA	↑(62%)		↑(42%)	
2-AG		**↓(25%)**	↑(21%)	**↓(33%)**
PGE2	↑(119%)			↑(62%)
PGF2*α*				

**Table:** (b)

Paced mating compared to chamber exposed				
	Hippocampus	Midbrain	Striatum	Brainstem
AEA	**↓(49%)**	**↓(44%)**	**↓(45%)**	**↓(52%)**
NAGly	**↓(57%)**	**↓(40%)**		**↓(35%)**
PEA	**↓(64%)**	**↓(39%)**	**↓(47%)**	**↓(41%)**
SEA	**↓(50%)**	**↓(25%)**		**↓(22%)**
OEA	**↓(53%)**	**↓(42%)**	**↓(45%)**	**↓(39%)**
DHEA	**↓(50%)**	**↓(32%)**	**↓(45%)**	**↓(28%)**
2-AG			**↓(24%)**	
PGE2	**↓(63%)**			
PGF2*α*	**↓(49%)**			

**Table:** (c)

Paced mating compared to standard mating				
	Hippocampus	Midbrain	Striatum	Brainstem
AEA		**↓(33%)**	**↓(48%)**	**↓(40%)**
NAGly		**↓(17%)**	↑(23%)	
PEA	**↓(44%)**	**↓(34%)**	**↓(40%)**	
SEA		**↓(29%)**	**↓(40%)**	
OEA	**↓(40%)**	**↓(38%)**	**↓(41%)**	
DHEA	**↓(32%)**	**↓(31%)**	**↓(40%)**	
2-AG			**↓(18%)**	**↓(25%)**
PGE2			**↓(59%)**	
PGF2*α*		**↓(33%)**	**↓(48%)**	**↓(40%)**
